# Marine Peptide-N6NH2 and Its Derivative-GUON6NH2 Have Potent Antimicrobial Activity Against Intracellular *Edwardsiella tarda in vitro* and *in vivo*

**DOI:** 10.3389/fmicb.2021.637427

**Published:** 2021-03-09

**Authors:** Huihui Han, Da Teng, Ruoyu Mao, Ya Hao, Na Yang, Zhenlong Wang, Ting Li, Xiumin Wang, Jianhua Wang

**Affiliations:** ^1^Gene Engineering Laboratory, Feed Research Institute, Chinese Academy of Agricultural Sciences, Beijing, China; ^2^Key Laboratory of Feed Biotechnology, Ministry of Agriculture and Rural Affairs, Beijing, China; ^3^Chinese Herbal Medicine Laboratory, Feed Research Institute, Chinese Academy of Agricultural Sciences, Beijing, China

**Keywords:** marine peptide, *Edwardsiella tarda*, intracellular activity, mechanism, macrophages

## Abstract

*Edwardsiella tarda* is a facultative intracellular pathogen in humans and animals. There is no effective way except vaccine candidates to eradicate intracellular *E. tarda*. In this study, four derivatives of marine peptide-N6NH2 were designed by an introduction of unnatural residues or substitution of natural ones, and their intracellular activities against *E. tarda* were evaluated in macrophages and in mice, respectively. The minimum inhibitory concentration (MIC) value of N6NH2 and GUON6NH2 against *E. tarda* was 8 μg/mL. GUON6NH2 showed higher stability to trypsin, lower toxicity (<1%) and longer post-antibiotic effect (PAE) than N6NH2 and other derivatives. Antibacterial mechanism results showed that GUON6NH2 could bind to LPS and destroyed outer/inner cell membranes of *E. tarda*, superior to N6NH2 and norfloxacin. Both N6NH2 and GUON6NH2 were internalized into macrophages mainly via lipid rafts, micropinocytosis, and microtubule polymerization, respectively, and distributed in the cytoplasm. The intracellular inhibition rate of GUON6NH2 against *E. tarda* was 97.05–100%, higher than that in case of N6NH2 (96.82–100%). In the *E. tarda*-induced peritonitis mouse model, after treatment with of 1 μmol/kg N6NH2 and GUON6NH2, intracellular bacterial numbers were reduced by 1.54- and 1.97-Log_10_ CFU, respectively, higher than norfloxacin (0.35-Log_10_ CFU). These results suggest that GUON6NH2 may be an excellent candidate for novel antimicrobial agents to treat infectious diseases caused by intracellular *E. tarda.*

## Introduction

*Edwardsiella tarda* is a Gram-negative bacterium that can infect a wide range of hosts, including humans and animals (Mohanty and Sahoo, 2007). As a human pathogen, *E. tarda* can cause gastroenteritis and parenteral diseases such as peritonitis, meningitis, and myonecrosis (Wang et al., 2005). Additionally, *E. tarda* is known to affect large amounts of freshwater and seawater fish, leading to serious economic losses (Matsuyama et al., 2005). Noticeably, *E. tarda* is a facultative intracellular bacterial pathogen with the ability to invade host phagocytes and non-phagocytes, which can survive and proliferate in macrophages, resulting in the persistence and recurrence of bacterial infections (Wuermeling, 1991; Ling et al., 2000; Srinivasa Rao et al., 2001; Okuda et al., 2006; Ishibe et al., 2008; Leung et al., 2012; Wang et al., 2013). Antibiotics are the primary drugs to prevent bacterial infection and are of great significance to human and animal health. However, antibiotics such as oxacillin, levofoxacin, garenoxacin, moxifoxacin, and oritavancin have poor bactericidal activity against intracellular bacteria due to their slow penetration rate and low concentration within eukaryotic cells. Therefore, traditional antibiotics must be used at a high enough extracellular concentration to achieve significant activity, which in turn may increase the problems of bacterial resistance and other side effects. Until now, there is no effective method except vaccine candidates to eradicate intracellular *E. tarda* in hosts (Xu and Zhang, 2014).

Based on the advantages of broad-spectrum activity, rapid killing rate and low drug resistance, antimicrobial peptides (AMPs) open a new way for the development of antimicrobial agents (Czaplewski et al., 2016). It has been demonstrated that some AMPs such as cathelicidin (Sonawane et al., 2011), GSK1322322 (Dubois et al., 2015), plectasin derivatives-MP1102/NZ2114/H2 (Brinch et al., 2010; Wang X. et al., 2018, 2019), and temporins (Crepin et al., 2020) exhibit potent intracellular activity against *Mycobacterium tuberculosis*, *Legionella pneumophila*, methicillin-susceptible/-resistant *Staphylococcus aureus*, and *Legionella pneumophila*, respectively. A marine peptide-NZ17074 (N1) is a variant of arenicin-3 (Tyr5→Asn, Tyr17→His) isolated from marine lugworm *Arenicola marina* and has strong intracellular antibacterial activity against Gram-negative bacteria (including *Escherichia coli*, *Salmonella*, *Pseudomonas aeruginosa*, etc.) and fungi (Yang et al., 2017). However, N1 has some toxicity to eukaryotic cells. To further improve the antibacterial activity of N1, C-terminal amidated N6 (N6NH2) exhibits low cytotoxicity and potent intracellular antibacterial activity against *Salmonella* than N1 (Li et al., 2018). However, N6NH2 is very susceptible to trypsin, which will limit its clinical application in the future.

In this study, four derived peptides of N6NH_2_ were designed by using natural residues (Val and Pro), unnatural residue (ornithine, Orn) and D-amino acids to improve the stability of N6NH2 toward protease and the antibacterial activity (Jia et al., 2017), while keeping the structural features (β-sheet) known to be important for the antimicrobial activity of peptides. The antibacterial activity, stability and toxicity of N6NH2 and its derivatives were evaluated *in vitro*, as well as their mode of action. The uptake and mechanism of N6NH2 and its derivatives were further elucidated and their intracellular activity against *E. tarda* was evaluated in RAW 264.7 macrophages and mice, respectively.

## Materials and Methods

### Design and Physicochemical Property of Peptides

The derivatives of N6NH2 were designed based on the replacement of L-type amino acids, Gly, and Arg by D-type ones, Pro, and Val or the addition of N,N,N’,N’-tetramethylguanidine and L-ornithine (Orn) at the N-terminus (Table 1). The physicochemical properties of the peptides were analyzed by HeliQuest^1^ and PepCalc.com^2^, respectively. GUON6NH2 was synthesized by WuXi AppTec (Wuhan, China) and other peptides were synthesized by Mimotopes (Wuxi, China). The purity of peptides was over 90%.

**TABLE 1:** Peptide design and their key physicochemical parameters.

**Name**	**Sequence^a^**	**Theoretical MW (Da)**	**Measured MW (Da)^b^**	**Net charge (+)**	**pI**	**H^c^**	**μHrel^d^**
N6NH2	GFAWNVCVYRNGVRVCHRRAN-NH2	2476.85	2476.8	5	11.64	0.375	0.2
DN6NH2	GFAWNVCVYRNGVRVCHRRAN-NH2	2476.85	2474.88	5	11.64	0.375	0.2
N6PNH2	GFAWNVCVYRNGVRVCHRPAN-NH2	2417.78	2415.81	4	10.91	0.457	0.25
V112N6NH2	VFAWNVCVYRNVVRVCHRRAN-NH2	2561.94	2559.04	5	11.64	0.491	0.286
GUON6NH2	Gu-OGFAWNVCVYRNGVRVCHRRAN-NH2	2631.08	2630.1	5	8.5	NP	NP

*NP, no predication. ^*a*^Gu denotes N,N,N’,N’-tetramethylguanidine, and O denotes L-ornithine. ^*b*^Molecular weight (MW) was measured by mass spectroscopy (MS). ^*c*^Hydrophobicity (H) values means the total hydrophobicity (sum of all residue hydrophobicity indices) divided by the number of residues, and they were calculated from http://heliquest.ipmc.cnrs.fr/cgi-bin/ComputParams.py. ^*d*^The relative hydrophobic moment (μHrel) of a peptide is its hydrophobic moment relative to that of a perfectly amphipathic peptide. This gives a better idea of the amphipathicity using different scales. A value of 0.5 thus indicates that the peptide has about 50% of the maximum possible amphipathicity were calculated from http://heliquest.ipmc.cnrs.fr/cgi-bin/ComputParams.py.*

### Circular Dichroism (CD) of N6NH2 and Its Derivatives

The secondary structure of peptides was investigated in ddH_2_O, trifluoroethanol (TFE), and sodium dodecyl sulfate (SDS) solutions by CD spectroscopy (Pavia et al., 2012). Peptides were dissolved in ddH_2_O, 20 mM SDS solutions or 50% TFE. The CD spectra of the peptides were measured on a MOS-450 spectropolarimeter (Bio-Logic, Grenoble, France) using a 1.0 mm path-length cuvette. The spectra of peptides were recorded from 180 to 260 nm at 25°C at a scanning speed of 100 nm/min with a step resolution of 2.0 nm and an integration time of 2 s. Data were analyzed using CDNN software.

### Antimicrobial Activity and Time-Killing Curve of N6NH2 and Its Derivatives

The antimicrobial activity of peptides was determined by the microbroth dilution method (Zhang et al., 2014). The bacterial strains were incubated in Mueller-Hinton (MH) medium at 28°C or 37°C overnight. Bacteria (1 × 10^5^ CFU/mL) were added into 96-well plates, followed by the addition of serial dilutions of peptides (from 0.0625 to 128 μg/mL). The plates were incubated at 37°C for 18–24 h. The minimal inhibitory concentration (MIC) value was determined as the lowest peptide concentration at which no bacterial growth was observed.

Time-killing curve of peptides was performed according to the previous method (Flamm et al., 2019). *E. tarda* was cultured to logarithmic phase, diluted in Mueller Hinton broth (MHB) (1 × 10^5^ CFU/mL) containing 1×, 2×, or 4× MIC of peptides, and cultured at 28°C (200 rpm). PBS was added as a control. Bacterial samples (100 μL) were removed at different time intervals (0, 0.5, 1, 1.5, 2, 4, 6, 8, 10, 12, 22, and 24 h) and were counted on MH solid plates. The time-killing curves of peptides were plotted. Bacterial cells treated with 2× MIC norfloxacin and without treatment were used as the positive and blank controls, respectively.

### Cytotoxicity, Hemolysis, and Stability of N6NH2 and Its Derivatives

The cytotoxicity of N6NH2 and its derivatives to RAW 264.7 cells was determined by MTT (Wang J. et al., 2018). Briefly, macrophage cells (5 × 10^3^ cells/well) were added into 96-well microtiter plates and incubated at 37°C for 24 h (5% CO_2_). A series of peptide solutions were then added into plates and incubated for 24 h. The cells without treatment were used as controls. The 3-(4,5-dimethylthiazol-2-yl)-2,5-diphenyl tetrazolium bromide 3-(4,5)-dimethylthiahiazo (-z-y1)-3,5-di-phenytetrazoliumromide (MTT) solution was added and incubated for 4 h. Finally, dimethyl sulfoxide (DMSO) was added into the plates and the absorbance was measured at 570 nm with a spectrophotometer. The cell survival was calculated using the following formula: Survival (%) = (Abs _control_ – Abs _treated sample_)/Abs _control_ × 100.

The hemolysis of peptides was evaluated by determining the amount of hemoglobin that was released from fresh mouse erythrocytes (Flamm et al., 2019). The blood cells were washed three times with 10 mM PBS (pH 7.4) and centrifuged for 10 min (1,500 rpm). Erythrocyte solution (8%, v/v) was mixed with peptide solutions (1:1) and incubated for 1 h at 37°C. The mixture was then centrifuged for 5 min (1,500 rpm), and the absorbance of supernatants was measured at 540 nm. Values of 0 and 100% hemolysis were determined in PBS and 0.1% triton X-100, respectively. The hemolysis of peptides was calculated using the following formula: Hemolysis (%) = [(Abs _treated sample_ – Abs _negative control_)/(Abs _positive control_ – Abs _negative control_)] × 100.

To determine effects of temperature on antibacterial activity, the peptides were incubated for 1 h at 4, 20, 40, 60, 80, and 100°C in PBS. The pH stability of N6NH2 and its derivatives was determined after 3 h incubation in 100 mM glycine-HCl buffer (pH 2.0), sodium acetate buffer (pH 4.0), sodium phosphate buffer (pH 6.0), Tris-HCl buffer (pH 8.0), or glycine-NaOH buffer (pH 10.0). In addition, the peptides were incubated for 4 h at 37°C in pepsin (3,000 U/mg, pH 2.0) and trypsin (250 U/mg, pH 8.0) (10:1, w/w) solutions, respectively. The antibacterial activity of peptides was estimated against *E. tarda* cells by an inhibition zone method (Zhang et al., 2014).

All tests were conducted in triplicate.

### Synergism of N6NH2 and Its Derivatives With Antibiotics

Combination of N6NH2, GUON6NH2 and antibiotics were tested by using the checkerboard titration method as described previously (Ejim et al., 2011; Zhang et al., 2014). MIC values of six kinds of traditional used antibiotics (ciprofloxacin, ofloxacin, enrofloxacin, norfloxacin, chloramphenicol, and kanamycin) to *E. tarda* were determined as the MIC assay described above. Different concentrations of antibiotics and antimicrobial peptides were added to each row and column of the 96-well cell culture plates to make the final concentration of 1/16–8 × MIC. After adding 180 μL of test bacteria solution to give a total volume in each well of 200 μL, the plates were incubated at 28°C for 18–24 h to observe the bacterial growth during the combination of drugs. Briefly, *E. tarda* (80 μL, 5 × 10^5^ CFU/mL) was incubated with 10 μL of single peptides or antibiotics or their combination in 96-well plates. PBS was used as a negative control. After incubation at 37°C overnight, the OD_600 nm_ value of bacterial cultures was measured with a microplate plate reader. The fractional inhibitory concentration index (FICI) is calculated as follows: FICI = FIC_A_ + FIC_B_, where FIC_A_ and FIC_B_ are the MICs of drug A in the combination divided by the MICs of drug A alone and the MICs of drug B in the combination divided by the MICs of drug B alone, respectively. FICI ≤ 0.5, 0.5 < FICI ≤ 1.0, 1.0 < FICI ≤ 4.0, and FICI > 4.0 were defined as a synergy, addition, indifference, and antagonism, respectively. The results were performed from at least three independent experiments.

### Post-antibiotic Effect (PAE) of Peptides and Antibiotic

*E. tarda* cells were incubated with peptides (1×, 2×, or 4× MIC) for 2 h, diluted, and cultured at 28°C and 200 rpm. An aliquot of samples (50 μL) were taken for CFU counts at different time intervals until bacterial cultures became turbid. Bacteria treated with the N6NH2, GUON6NH2 or vancomycin were used as the positive control; bacteria without treatment were used as the blank control. The PAE was calculated using the following formula: PAE = T–C, where T is the time for the CFU counts to increase by 10-fold above the count observed immediately after drug removal, and C is the corresponding time for the untreated control (Oh et al., 2019).

### The Ability of N6NH2 and Its Derivatives to Bind to LPS

The BODIPY’-TR-cadaverine (BC) probe was used to determine the ability of peptides to bind to LPS (Wang et al., 2020). 0.5 μM LPS was incubated with 5 μM BC in 50 μM Tris buffer (pH 7.4) for 4 h at 37°C. Subsequently, the peptides were added into the mixture and the final concentrations of peptides ranged from 0.049 to 50 μM. Ampicillin was used as the negative control. Changes in fluorescence were measured by a fluorescence spectrophotometry at room temperature (excitation/emission, 580/620 nm).

### Membrane Permeabilization Ability of N6NH2 and Its Derivatives Against *E. tarda*

The outer membrane permeabilization ability of the peptides was determined using the fluorescent dye 1-N-Phenylnapthylamine (NPN) assay (Teng et al., 2014). Mid-log phase *E. tarda* cells were collected by centrifugation and washed twice and then suspended in HEPES buffer (pH 7.4) to an OD_600nm_ of 0.4. Cell suspensions and NPN solutions (10 μM) were added into 96-well black plates, followed by the addition of peptide solutions (1×, 2×, and 4× MIC, respectively). Fluorescence intensity was recorded with a microplate reader (excitation/emission, 328/438 nm). The cells treated PBS and without treatment were used as negative and blank controls, respectively.

Mid-log phase *E. tarda* cells (1 × 10^8^ CFU/mL) were incubated with or without 1× MIC peptides solutions at 37°C for 5, 15, and 30 min, respectively. After washing twice with PBS, bacterial cells were then incubated with 50 μg/mL propidium iodide (PI) for 15 min and analyzed using a FACS Calibur Flow Cytometer (BD, United States) (Stearns-Kurosawa et al., 2011).

### Scanning Electron Microscopy (SEM) Observations

To further characterize the bactericidal effects of the peptides, scanning electron microscopy (SEM) and transmission electron microscopy (TEM) were used to visualize morphological changes (Yang et al., 2019). *E. tarda* was cultured to logarithmic growth stage (10^8^ CFU/mL) and diluted with 0.01 M PBS (pH 7.4) to OD_600 nm_ = 1. *E. tarda* cells were treated with 4× MIC peptides at 37°C for 2 h and the untreated cells were used as the control. Briefly, after centrifugation, the bacterial cells were washed three times with 0.1 M PBS (pH 7.2) and fixed overnight at 4°C with 2.5% glutaraldehyde. After washing again, the cells were fixed with 1% osmium tetroxide (OsO_4_) for 2 h and dehydrated in a graded ethanol series (50%–70%–85%–95% × 2–100%, × 2) for 15 min, respectively. The samples were then dried by CO_2_, sputtered with gold-palladium, and observed on a QUANTA200 SEM (FEI, Philips, Netherlands).

### Transmission Electron Microscopy (TEM) Observations

*E. tarda* cells were treated peptides before fixation as described above. The bacteria were washed with 0.01 M PBS (pH 7.4) for five times, 7 min each time, and fixed with 1% osmium acid buffer (4°C) for 1 h. After dehydration with a graded acetone series (30%–50%–70%–85%–95%–100% × 3), the cells were immersed in mixtures of acetone and resin (3:1, 1:1 and 1:3, respectively) for 40 min and in a pure epoxy resin overnight. The samples were then embedded in embedding medium and polymerized at 45°C for 3 h and at 65°C for 24 h, respectively. The sections were prepared using an ultramicrotome, stained with 1% uranyl acetate, and visualized by a JEM1400 (JEDL, Tokyo, Japan).

For immuno-TEM, *E. tarda* cells in the exponential phase (10^8^ CFU/mL) were treated with 2× MIC N6NH2 or GUON6NH2 at 37°C for 2 h or untreated as control. The cells were fixed with 4% formaldehyde at room temperature for 1 h and at 4°C overnight and then immersed in 1% formaldehyde. The samples were dehydrated in a graded ethanol series (30–100%, 4°C, 5 min), infiltrated with LR Gold resin (25–100%, 4°C, 1 h), and polymerized by at 52°C for 48 h. 70 nm sections were cut using Leica EM UC7 and collected on nickel grids with Formvar/carbon film. The sections were blocked at 2% BSA (Jackson ImmunoResearch) and incubated with murine anti-biotin antibodies at 1:40 dilution at room temperature for 1 h. After washing with PBS (containing 0.1% cold fish gelatin), the sections were incubated with 12 nm gold-labeled anti-mouse secondary antibody (Jackson ImmunoResearch) at room temperature for 1 h, washed with PBS, and fixed by 1% glutaraldehyde. After washing with ddH_2_O, the samples were stained with 2% uranyl acetate at room temperature for 30 min and imaged using a TEM H-7650 at 80 kV (Möbius et al., 1999).

### Fluorescence Microscopy

The site of action of the peptides on *E. tarda* was preliminarily determined by super resolution fluorescence microscopy using FITC-labeled peptides and PI, as previously described (Wang J. et al., 2019). *E. tarda* was cultured to logarithmic growth stage (10^8^ CFU/mL), centrifuged at 5,000 rpm for 5 min and diluted with 0.01 M PBS (pH 7.4) to OD_600 nm_ = 1. In brief, *E. tarda* was treated with FITC-labeled peptides (1× or 4× MIC) at 37°C for 30 min. Then, the bacterial cells were washed three times with PBS, incubated with PI (10 μg/mL) at 4°C for 15 min, and washed again; 5 μL of bacterial suspension was coated on a slide and sealed with a cover slide. The samples were observed using a DeltaVision OMX SR system (GE Healthcare, United States) at excitation wavelengths of 488 and 535 nm for PI and FITC, respectively. Cells without peptides served as controls.

### Intracellular Replication With *E. tarda*

RAW 264.7 cells were infected with *E. tarda* and extracellular *E. tarda* was killed by adding gentamicin (1000 μg/ml) to the plate, followed by incubation at 37°C for 30 min. The cells were washed three times with PBS, and cultured in DMEM for 0, 6, 12, 18, and 24 h, respectively. At each time point, 500 μL 0.1% triton X-100 was added to the plate, and the samples were collected and diluted; the bacteria were counted as previously described (Wang et al., 2017). The experiment was performed in triplicate.

### Uptake and Mechanism of N6NH2 and GUON6NH2 in RAW 264.7 Macrophages

RAW 264.7 macrophages (1.5 × 10^4^ cells) were cultured for 24 h and incubated with FITC-labeled N6NH2 and GUON6NH2 (5× and 25× MIC) for 18 h at 37°C. After incubation, the cells were then washed with PBS and stained with wheat germ agglutinin (WGA)-conjugated Alexa Fluor 555 (5 μg/mL, Invitrogen) and Hoechst 33342 (5 μg/mL, Invitrogen) for membrane and nuclear staining, respectively, for 10 min before confocal microscopy (Duchardt et al., 2009; Kamaruzzaman et al., 2016).

To quantify peptide uptake, RAW 264.7 macrophages were grown in 12-well plates (2.5 × 10^5^ cells/mL, 750 μL/well) for 24 h. Then, the cells were treated with FITC-N6NH2 and FITC-GUON6NH2 (1, 5, 10, 25, and 50× MIC) for 18 h and washed with PBS. To avoid the influence of membrane-bound FITC-labeled peptides, the cells were incubated with 0.04% trypan blue in PBS for 15 min and the fluorescence intensity was analyzed using a FACS Calibur Flow Cytometer (BD, United States) (Duchardt et al., 2009).

To determine the effects of endocytosis inhibitors on peptide uptake, RAW 264.7 macrophages were pretreated with 3 mM amiloride, 5 mM methyl-β-cyclodextrin (MβCD), 20 μM nocodazole, and 6 μg/mL chlorpromazine for 1 h at 37°C. The cells were then treated with FITC-N6NH2 and FITC-GUON6NH2 at 37°C for 4 h. The cells treated with the FITC-labeled peptides were used as a positive control. Finally, the cells were mixed with 0.04% trypan blue and florescence within cells were measured using flow cytometry (Gomarasca et al., 2017).

### *In vitro* Efficacy of N6NH2 and GUON6NH2 Against *E. tarda* in RAW 264.7 Cells

For intracellular activity, RAW 264.7 macrophages (2.5 × 10^5^ cells/mL) were cultured for 24 h in DMEM with 10% FBS (without antibiotics) (Edwards and Massey, 2011). Meanwhile, mid-log phase *E. tarda* were collected by centrifugation, diluted to a concentration of 2.5 × 10^8^ CFU/mL in DMEM with 10% FBS (without antibiotic), and incubated with RAW 264.7 macrophages for 0.5 h. Gentamicin (1,000 μg/mL) was then added into the cells and incubated for 30 min to remove extracellular bacteria. After washing twice with PBS, RAW 264.7 macrophages were treated with different concentrations of N6NH2 and GUON6NH2 (1, 5, 10, 25, and 50× MIC) for 18 h, washed again, and lysed with Hanks buffered saline solution (0.1% bovine serum albumin and 0.1% Triton-X). The numbers of intracellular bacteria were counted at 0 and 18 h, respectively.

### *In vivo* Efficacy of N6NH2 and GUON6NH2 in a Mouse Peritonitis Model

To establish a peritonitis mouse model, six-week-old female ICR mice (five mice/group) were intraperitoneally injected with *E. tarda* (1 × 10^9^ CFU/mL, 0.5 mL). The infected mice were followed by the intraperitoneal injection of N6NH2, GUON6NH2 or norfloxacin (1 μmol/kg of body weight, 0.2 mL) at 1 h postinfection, respectively. Mice injected with only bacteria or PBS were used as negative or blank controls. The survival of mice was recorded daily for 5 days.

The mice were intraperitoneally infected with *E. tarda* (1 × 10^9^ CFU/mL, 0.5 mL) and then injected with N6NH2 and GUON6NH2 (1 μmol/kg) or PBS (the blank control) in the same way at 1 h after infection. The mice were then killed at 24 h after treatment, and peritoneal fluids were obtained by washing with 5 mL of ice-cold PBS. The total number of *E. tarda* in the fluids was determined before any further procedures. For intracellular bacteria quantification, the other fraction was centrifuged for 5 min at 4°C (1,000 rpm). The cells were collected, treated with 1,000 μg/mL gentamicin for 30 min at 37°C to kill extracellular bacteria, and washed with ice-cold PBS twice to remove extracellular gentamicin. Subsequently, the cells were lysed and the bacteria were counted as described above for the *in vitro* experiment (Kim et al., 2011).

## Results

### Design and Characterization of N6NH2 and Its Derivatives

DN6NH2 was generated by replacement of L-type amino acids of N6NH2 (except Gly) with D-type ones; N6PNH2 was obtained by substitution of Arg in position 19 with Pro; Gly substitution with Val in position 1 and 12 led to V112N6NH2; GUON6NH2 was generated by addition of N,N,N’,N’-tetramethylguanidine and L-Orn to N-terminus of N6NH2. As shown in Table 1, physicochemical properties (MW, isoelectric point (pI), charge, hydrophilicity) of DN6NH2 were almost equal to those of its parent peptide-N6NH2. The net charge of N6PNH2 (+4) was less than that of N6NH2 and other derivatives (+5), but its hydrophobicity increased from 0.375 to 0.457; V112N6NH2 had higher hydrophobicity than N6NH2 and other derivatives.

### Structures of N6NH2 and Its Derivatives Analyzed by CD

The anionic surfactant SDS and TFE are often used to mimic the cell membrane environment. To analyze the structural features of N6NH2 and its derivatives, the CD spectra of peptides were measured in ddH_2_O, 20 mM SDS and 50% TFE, respectively. As shown in Supplementary Figure 1 and Supplementary Table 1, the secondary structure of N6NH2, N6PNH2, and V112N6NH2 in ddH_2_O was characterized by random coils (34.57–42.76%), antiparallel strands (15.17–30.36%) or β-turns (22.21–42.76%), with a negative minimum at 200 nm. However, GUON6NH2 showed a significant decrease in the proportion of anti-parallel strands (1.04%). DN6NH2 mainly contained anti-parallel chains and random coils, with a positive maximum at 200 nm. In SDS solution, N6NH2 had higher antiparallel chain (63.52%) than N6PNH2 (17.78%) and V112N6NH2 (14.77%); DN6NH2 changed from antiparallel strands to β-turns (38%) and random coils (52.22%). The secondary structures of N6PNH2, V112N6NH2 and GUON6NH2 in 50% TFE were characterized predominantly by β-turns, random coils, anti-parallel chains or β-turns, with a negative dichroic band at approximately 205 nm, and a positive maximum at 180 nm (strong) and 230 nm (weak).

### Antimicrobial Activity and Time-Killing Curve of N6NH2 and Its Derivatives

As shown in Table 2, N6NH2 and its derivatives had stronger antibacterial activity against Gram-negative bacteria such as *Escherichia coli*, *Salmonella* and *E. tarda* than Gram-positive bacteria. N6NH2, DN6NH2 and GUON6NH2 exhibited the strongest antimicrobial activity against Gram-negative and Gram-positive bacteria with MIC values ranging from 2 to 32 μg/mL. The MIC values of N6PNH2 and V112N6NH2 against *E. coli*, *Salmonella* and *E. tarda* were 4–8, 2–32, and 8–32 μg/mL, respectively. The MIC value of N6NH2, DN6NH2 and GUON6NH2 against *E. tarda* was 8 μg/mL, indicating their similar antibacterial activity.

**TABLE 2:** MIC values of N6NH2 and its analogs.

**Species and Strains**	**MIC (μg/mL)**				
	**N6NH2**	**N6PNH2**	**DN6NH2**	**V112N6NH2**	**GUON6NH2**
**Gram-negative bacteria**					
*Escherichia coli* CVCC195^a^	4	8	8	8	4
*E. coli* CVCC1515^a^	2	4	4	4	2
*E. coli* CVCC25922^a^	4	8	8	8	4
*E. coli* CVCCO157^a^	4	8	4	8	4
*Salmonella typhimurium* CVCC533^a^	4	32	16	16	8
*S. typhimurium* ATCC14028^c^	4	16	8	16	4
*Streptococcus enteritidis* CVCC3377^a^	2	2	2	2	2
*Salmonella pullorum* CVCC1802^a^	4	8	8	8	4
*S. pullorum* CVCC1789^a^	8	16	16	16	8
*Edwardsiella tarda*	8	32	8	32	8
**Gram-positive bacteria**					
*Staphylococcus aureus* ATCC43300^c^	16	>64	4	32	16
*S. aureus* ATCC546^c^	16	>64	16	16	16
*S. aureus* ATCC25923^c^	32	>64	8	32	32
*Staphylococcus hyicus* 437-2	32	>64	8	32	32
*S. hyicus* 15095	8	64	4	16	4

*^*a*^China Veterinary Culture Collection Center (CVCC); ^*b*^China Center of Industrial Culture Collection (CICC); ^*c*^American Type Culture Collection (ATCC). Data were representative of three independent experiments.*

The geometric mean (GM) of MICs of peptides against Gram-negative and Gram-positive bacteria was calculated, and the result was shown in Table 3. Among these peptides, the GM MIC value of GUON6NH2 was 4.8 μg/mL, which was almost equal to N6NH2 (4.4 μg/mL), indicating their similarly potent antimicrobial activity. However, N6PNH2 with the largest GM MIC value of 13.4–115.2 μg/mL displayed the lowest activity, which may be related to less net charges. Moreover, N6PNH2 showed the worst cell selectivity at a therapeutic index (TI) of 2.22–19.1. For Gram-negative bacteria, the TI value of GUON6NH2 (53.33) was very close to N6NH2 (58.18), while for positive bacteria, the TI value of GUON6NH2 (12.8) was slightly higher than that of N6NH2 (12.31), indicating their huge therapeutic potential. For Gram-positive bacteria, DN6NH2 had the highest TI value and the best therapeutic potential.

**TABLE 3:** The MHC, GM, and TI values of N6NH2 and its derivatives.

**Peptide**	**MHC^a^ (μg/mL)**	**GM^b^ (μg/mL)**			**TI^c^**		
		**Gram-negative bacteria**	**Gram-positive bacteria**	**ALL**	**Gram-negative bacteria**	**Gram-positive bacteria**	**ALL**
N6NH2	>128	4.4	20.8	9.87	58.18	12.31	25.94
DN6NH2	>128	8.2	8	8.13	31.22	32	31.49
N6PNH2	>128	13.4	115.2	47.33	19.1	2.22	5.41
V112N6NH2	>128	11.8	25.6	16.4	21.69	10	15.61
GUON6NH2	>128	4.8	20	9.87	53.33	12.8	25.94

*^a^MHC is the minimum hemolytic concentration that caused 10% hemolysis of hRBCs. Data are representative of three independent experiments. When no detectable hemolytic activity was observed at 128 μg/mL, a value of 256 μg/mL was used to calculate the therapeutic index (TI).*

*^b^The geometric means (GM) of the peptide MICs against bacteria was calculated. When no detectable antimicrobial activity was observed at 64 μg/mL; a value of 128 μg/mL was used to calculate the GM.*

*^c^TI is calculated as MHC/GM. Larger values indicate greater cell selectivity.*

The bactericidal kinetics showed that similar to N6NH2, 2× or 4× MIC of V112N6NH2 and GUON6NH2 completely killed *E. tarda* within 6 h, which were more rapid than norfloxacin, N6PNH2, and DN6NH2 (Supplementary Figure 2). In the norfloxacin treatment group, the bacterial count at 2× MIC tended to decrease after 2 h and the bacteria re-grew at 12 h.

### Cytotoxicity, Hemolysis, and Stability of N6NH2 and Its Derivatives

**FIGURE 1:**
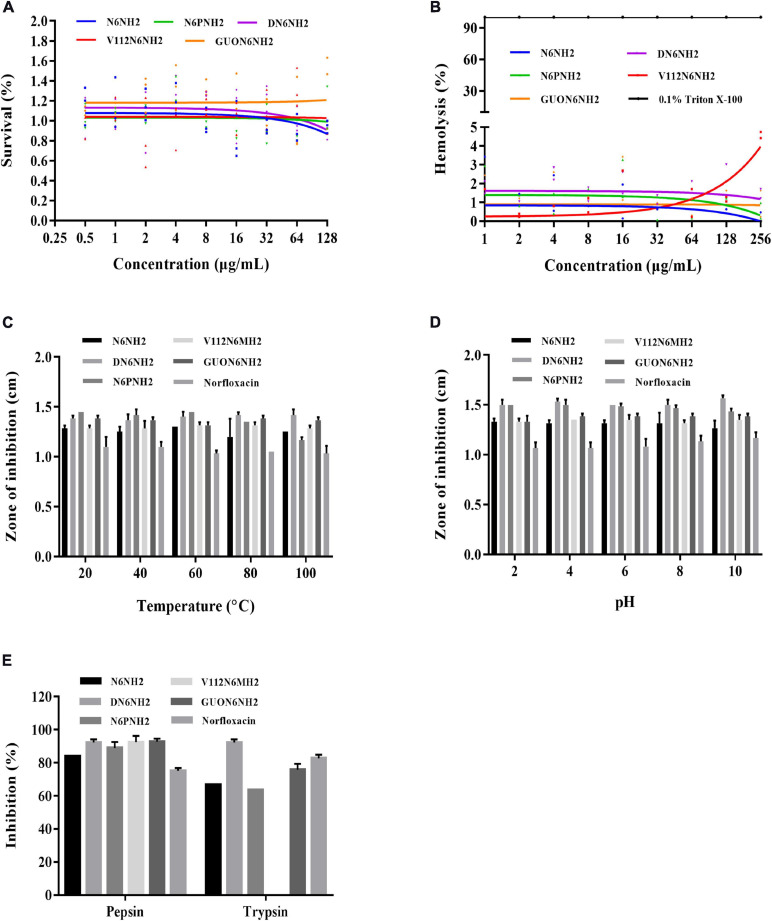
Hemolysis, cytotoxicity, and stability of N6NH2 and its derivatives. **(A)** Cytotoxicity of the peptides against RAW 264.7 cells. N6PNH2, V112N6NH2, and GUON6NH2 showed lower cytotoxicity than N6NH2 and DN6NH2. **(B)** Hemolytic activity of the peptides against murine erythrocytes. Derivatives of N6NH2 have very low or no hemolytic activity. **(C–E)** The effects of temperature **(C)**, pH **(D)**, and proteases **(E)** on the antibacterial activity of the peptides *in vitro* against *E. tarda*. The results are given as the mean ± SEM (*n* = 3). All peptides showed high stability toward temperature and pH. GUON6NH2 was more resistant to trypsin than N6NH2 and V112N6NH2, inferior to DN6NH2.

The cytotoxicity of peptides in murine peritoneal RAW 264.7 macrophage cells was determined by MTT assay. As shown in Figure 1A, the cell survival of N6PNH2, V112N6NH2 and GUON6NH2 at a concentration range of 64–128 μg/mL was higher than that of N6NH2 and DN6NH2, indicating their lower cytotoxicity than N6NH2 and DN6NH2. As shown in Figure 1B, at a concentration of 256 μg/mL, the hemolysis of DN6NH2, N6PNH2, V112N6NH2, and GUON6NH2 was 1.46, 0.56, 4.58, and 0.93%, respectively, higher than that of their parent peptide-N6NH2 (0.19%), indicating that these derivatives have very low or no hemolytic activity against murine erythrocytes.

High stability of drugs in complex environments is a prerequisite for the clinical application of AMP. Therefore, we evaluated the stability of the peptide to temperature, pH, and protease (Figures 1C–E). After exposure to different temperatures (20–100°C) for 1 h and pH values (pH 2–10) for 4 h, all peptides retained their inherent antibacterial activity against *E. tarda*, superior to norfloxacin. Additionally, similar to N6NH2, the derived peptides were highly resistant to pepsin, but sensitive to trypsin (Figure 1E). V112N6NH2 was completely intolerant to trypsin. GUON6NH2 was more resistant to trypsin than N6NH2, inferior to DN6NH2, and the enzyme stability of other peptides was lower than that of norfloxacin.

Based on the above results, both N6NH2 and GUON6NH2 were used to evaluate *in vitro* and *in vivo* efficacy due to their potent activity, low toxicity, and high stability.

### Synergism and PAE of N6NH2 and GUON6NH2

**FIGURE 2:**
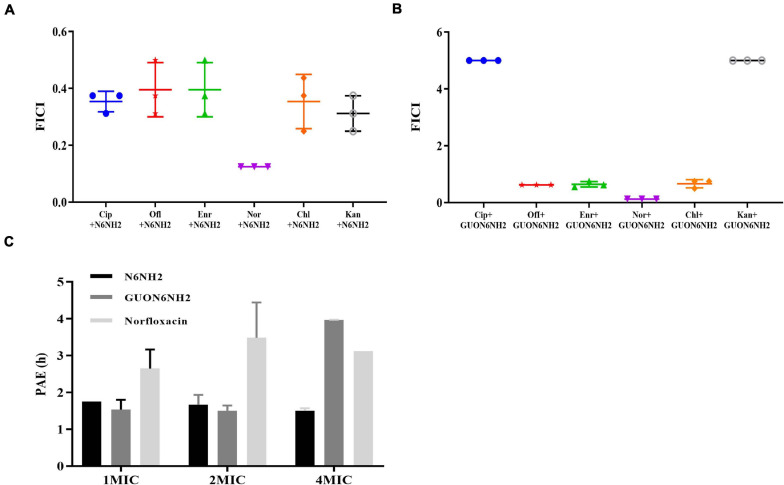
*In vitro* efficacy of N6NH2 and GUON6NH2. **(A,B)** Combination of peptide N6NH2 **(A)** and GUON6NH2 **(B)** with antibiotics against *E. tarda*, ciprofloxacin (Cip), ofloxacin (Ofl), enrofloxacin (Enr), norfloxacin (Nor), chloramphenicol (Chl), and kanamycin (Kan), respectively. **(C)** PAEs of N6NH2, GUON6NH2, and norfloxacin against *E. tarda*. The results are given as the mean ± SEM (*n* = 3).

The antibacterial activity of N6NH2 was synergistic with ciprofloxacin (FICI values of 0.3125), ofloxacin (FICI values of 0.3125), enrofloxacin (FICI values of 0.3125), norfloxacin (FICI values of 0.125), chloramphenicol (FICI values of 0.25), and kanamycin (FICI values of 0.25), respectively (Figure 2A and Supplementary Table 2). GUON6NH2 exhibited antagonism effects with ciprofloxacin and kanamycin (FICI values > 4), but additive effects with ofloxacin and enrofloxacin (FICI value of 0.625 and 0.5625, respectively); GUON6NH2 showed a synergistic effect with norfloxacin and chloramphenicol (FICI values of 0.125 and 0.5, respectively) (Figure 2B and Supplementary Table 2).

The efficacy of antibiotics is an important indicator of the frequency of medication (Zhanel and Craig, 1994). The PAE of N6NH2 at 1×, 2×, and 4× MIC was 1.75, 1.67, and 1.5 h, respectively; the PAE of GUON6NH2 was 1.54, 1.51, and 3.97 h, respectively, while the PAE of norfloxacin was 2.65, 3.48, and 3.12 h, respectively. At the same dose of 4× MIC, the PAE of GUON6NH2 to *E. tarda* was 2.65 and 1.27 times longer than that of N6NH2 and norfloxacin, respectively (Figure 2C).

### N6NH2 and Its Derivatives Bound to LPS

**FIGURE 3:**
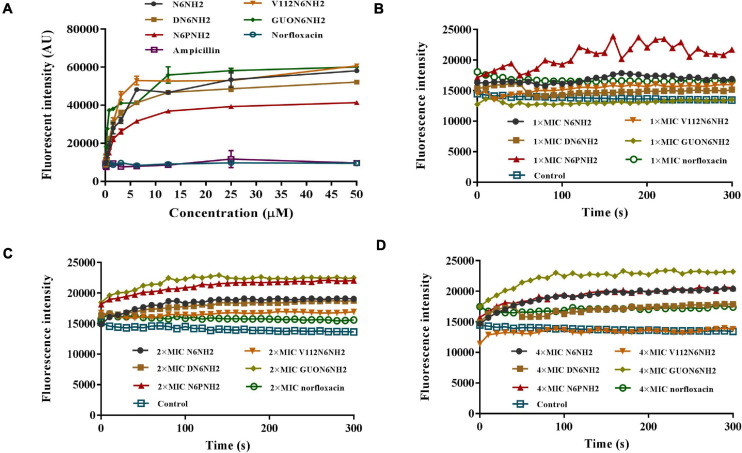
Interaction of N6NH2 and its derivatives with LPS and outer membrane of *E. tarda*. **(A)** Interaction of peptides with LPS (ability of LPS to displace a peptide-bound BC probe). All peptides showed a strong binding affinity for LPS. **(B–D)** Interaction of peptides with outer membrane of *E. tarda*. Bacterial cells were treated with 1× **(B)**, 2× **(C)**, and 4× MIC **(D)**, respectively, and observed with a microplate reader. GUON6NH2 exhibited a stronger ability to penetrate bacterial outer membrane than other peptides.

To reveal the mechanism of action of N6NH2 and its derivatives, the BC substitution method was used to evaluate the ability of the peptides to bind to LPS. As shown in Figure 3A, both ampicillin and norfloxacin did not replace BC probe to bind to LPS; the peptides showed a strong binding affinity for LPS in a concentration-dependent manner. The binding capacity in order was as follows: V112N6NH2 > GUON6NH2 > N6NH2 > DN6NH2 > N6PNH2.

### Mechanism of N6NH2 and Its Derivatives Against *E. tarda*

The NPN fluorescent dye was used to measure the permeability of the peptide to the outer membrane. N6NH2 and its derivatives quickly penetrated the outer membrane of *E. tarda* within 1 min (Figures 3B–D). All peptides induced an increase in NPN florescence in *E. tarda* cells in a time- and concentration-dependent manner. GUON6NH2 exhibited a stronger ability to penetrate the outer membrane of *E. tarda* than other peptides.

The fluorescent dye PI is blocked outside the intact cell membrane, but can penetrate the damaged cell membrane and insert nucleic acid (Wang et al., 2020). The fluorescence intensity of PI indicates the integrity of the cell membrane. As shown in Supplementary Figure 3, in the absence of peptides, 99% of cells showed no PI staining, indicating the intact cell membranes. After treatment with peptides for 5–30 min, the penetration rates of N6NH2, N6PNH2, and GUON6NH2 were 0.99–1.71, 0.42–2.20, and 0.78–2.84%, respectively, indicating that they weakly disrupt the inner membrane of *E. tarda*. Comparably, after treatment with DN6NH2 and V112N6NH2 for 5–30 min, the penetration rates were 1.3–16.1 and 0.85–30.1%, respectively, higher than those of other peptides. This result indicated that DN6NH2 and V112N6NH2 more potently penetrate the inner membrane of *E. tarda* than other peptides.

### Morphological Changes Observed by Electron Microscopy After Treatment With N6NH2 and Its Derivatives

**FIGURE 4:**
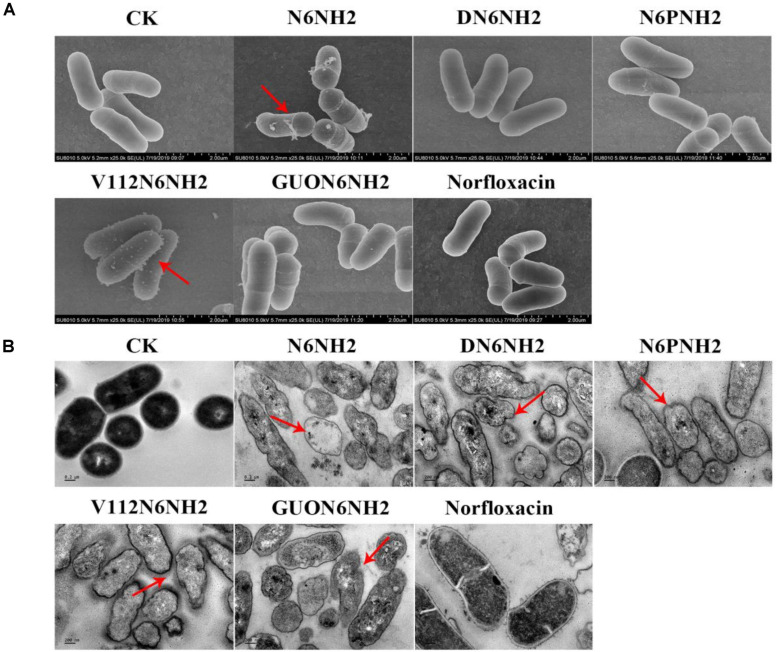
Effects of N6NH2 and its derivatives on the cell morphology and ultra-structures of *E. tarda.* Bacteria in mid-logarithmic growth phases were treated with peptides or antibiotic at 4× MIC for 2 h. **(A)** SEM images of *E. tarda* cells treated with peptides or norfloxacin. N6NH2, V112N6NH2, and GUON6NH2 induced some protrusions on the cell surfaces, but DN6NH2 and norfloxacin hardly changed the bacterial cell structures. **(B)** TEM images of *E. tarda* cells treated with peptides or norfloxacin. Deformed cell morphology, leakage of contents and heterogeneous cytoplasmic electron density were observed in bacterial cells with peptides; there was no changes in norfloxacin-treated cells.

The cells were treated with 4× MIC peptides or norfloxacin for 2 h, and the morphological changes in the cells were observed by SEM. As shown in Figure 4A, the untreated cells showed intact smooth surfaces. However, after treatment with N6NH2, V112N6NH2, and GUON6NH2, some protrusions were observed on the cell surfaces. In the DN6NH2- and norfloxacin-treated groups, there was no significant change in the bacterial cell structures.

The effects of peptides on the ultrastructures of *E. tarda* cells were observed using TEM. As shown in Figure 4B, in the untreated group, the cells exhibited normal cell morphology, intact cell membrane and uniform cytoplasmic electron density. After exposure to 4× MIC peptides for 2 h, it appeared to deformed cell morphology, leakage of cellular contents, and heterogeneous cytoplasmic electron density in *E. tarda*. However, in the norfloxacin-treated group, there was no significant change in bacterial cells.

Fluorescence microscopy was also used to analyze the effect of N6NH2 and GUON6NH2 on bacterial cells. As shown in Supplementary Figure 4, the green fluorescence completely covered the bacterial cells after treatment with FITC-peptides. Meanwhile, it was found that the green fluorescence was highly overlapped with red fluorescence-PI, indicating that N6NH2 and GUON6NH2 can enter *E. tarda* cells.

Immuno-TEM was used to further determine the effects of biotin-labeled peptides on the ultrastructures of *E. tarda*. As shown in Supplementary Figure 5, both biotin-labeled N6NH2 and GUON6NH2 were uniformly distributed in the cytoplasm of *E. tarda*, but overall cell morphology and the cell membrane of *E. tarda* had no significant change.

### The Intracellular Proliferation Curve of *E. tarda*

**FIGURE 5:**
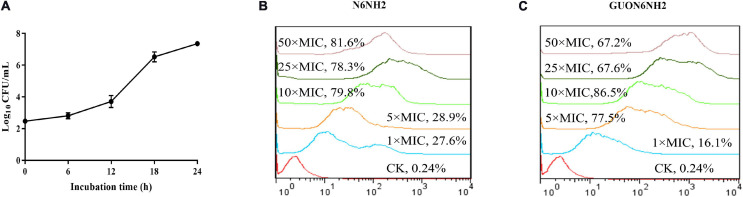
Proliferation of *E. tarda* and internalization of N6NH2 and GUON6NH2 in RAW 264.7 cells. **(A)** Proliferation of *E. tarda*. RAW 264.7 cells were infected with *E. tarda* for 0.5 h, incubated with gentamicin, and lysed with 1% triton-X-100. The bacteria were counted at 0, 6, 12, 18, and 24 h, respectively. The results are given as the mean ± SEM (*n* = 3). **(B,C)** FACS analysis of cellular internalization of FITC-labeled peptides in RAW 264.7 cells. The cells were incubated for 18 h with 1–50 × MIC FITC-N6NH2 **(B)** and FITC-GUON6NH2 **(C)** for 18 h at 37°C prior to washing and quantification of peptide uptake. The uptake of GUON6NH2 by macrophages is significantly greater than that of N6NH2.

After infection with *E. tarda* for 30 min, the extracellular bacteria were killed by gentamicin and macrophages were cultured to different time points to detect the number of intracellular bacteria. The results showed that after infection with *E. tarda* for 6–24 h, the number of intracellular bacteria increased from 2.52 Log_10_ CFU to 7.36 Log_10_ CFU (Figure 5A) in a time-dependent manner, suggesting that *E. tarda* can survive and proliferate in the RAW 264.7 cells.

### Uptake and Mechanism of N6NH2 and GUON6NH2 in RAW 264.7 Macrophages

The prerequisite for the elimination of intracellular *E. tarda* by antibacterial agents is that the drug effectively enters the host cells (Kuriakose et al., 2013; Lei et al., 2013). RAW 264.7 cells were treated with peptides and analyzed by flow cytometry (Floren et al., 2011). The results showed that after treatment with 1–10× MIC N6NH2 or GUON6NH2, the percentages of florescent cells were 27.6–79.8 and 16.1–86.5%, respectively (Figures 5B,C). Percentages of florescent cells slightly decreased after treatment with higher concentrations of peptides (except 50× MIC N6NH2). Moreover, both N6NH2 and GUON6NH2 entered macrophages in a concentration-dependent manner. These data implied that the uptake of GUON6NH2 by macrophages is significantly greater than that of N6NH2.

**FIGURE 6:**
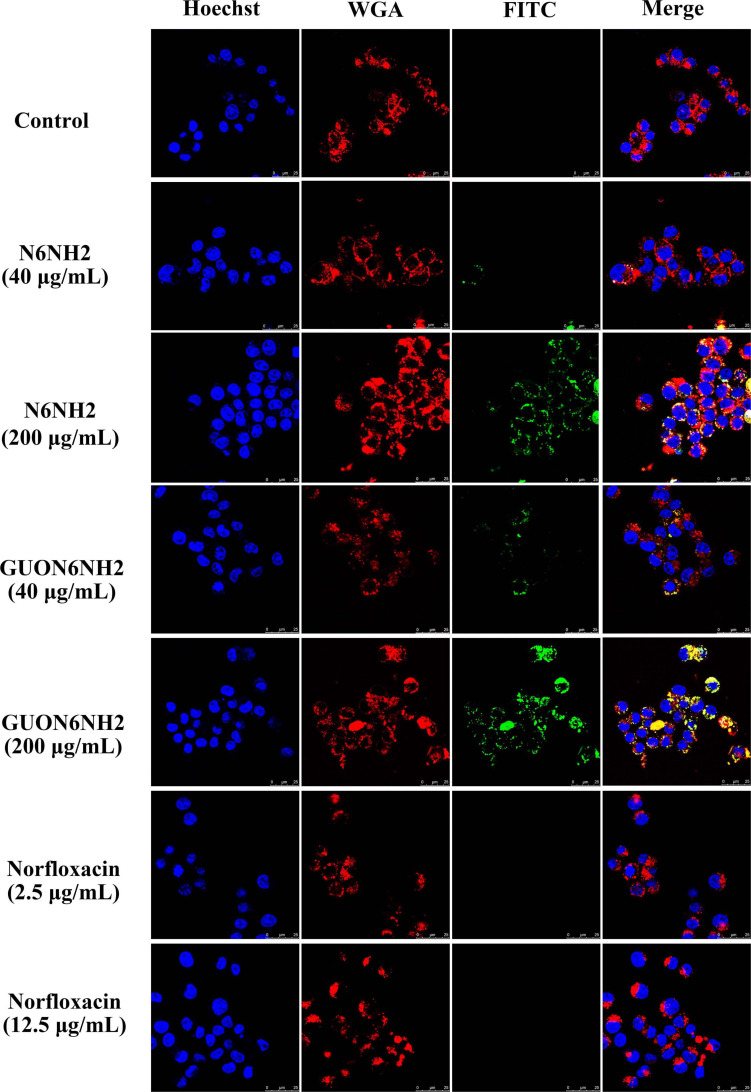
Cellular distribution of N6NH2 and GUON6NH2 in RAW 264.7 cells. Cells were incubated with 40 or 2.5 (5× MIC) and 200 or 12.5 μg/mL (25× MIC) FITC-labeled peptides or antibiotic at 37°C for 18 h before washing and analysis by confocal microscopy. Cell membrane and nucleus were stained with WGA-conjugated Alexa Fluor 555 (red) and Hoechst 33342 (blue), respectively. FITC-N6NH2 and GUON6NH2 inside cells displayed green fluorescence and were uniformly distributed in the cytoplasm. GUON6NH2 showed higher uptake than N6NH2, but FITC-labeled norfloxacin was not able to enter the cells.

To further determine uptake and subcellular distribution of peptides, RAW 264.7 cells were treated with peptides and observed using confocal microscopy. As shown in Figure 6, both FITC-labeled N6NH2 and GUON6NH2 could enter RAW 264.7 cells. With the increase in the concentration of the peptides (ranging from 40 to 200 μg/mL), the florescent intensity of FITC-labeled N6NH2 and GUON6NH2 was extremely enhanced in cells. In addition, the fluorescence intensity of GUON6NH2 was considerably higher than that of N6NH2, indicating higher uptake of GUON6NH2. Both peptides were uniformly distributed in the cytoplasm in granular form. Comparably, no green fluorescence was observed in the cells treated with FITC-labeled norfloxacin, indicating that FITC-labeled norfloxacin was not able to enter the cells.

**FIGURE 7:**
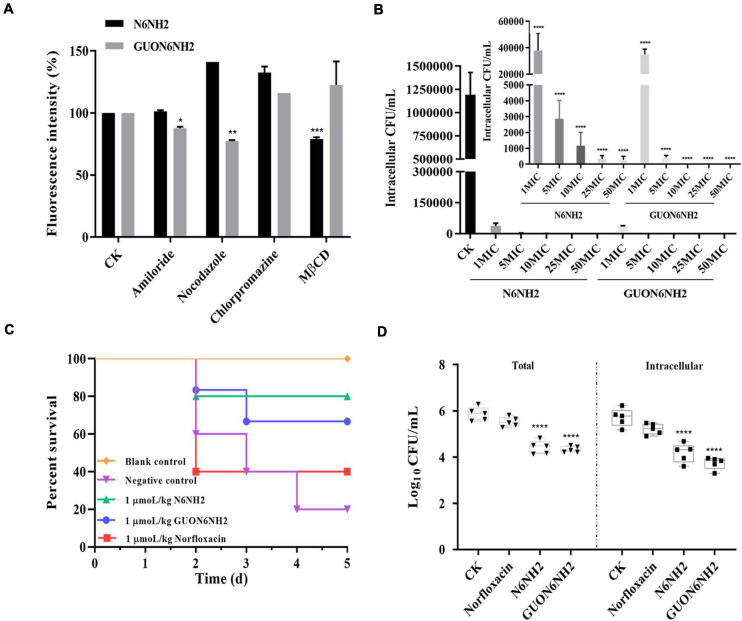
Uptake mechanism and intracellular activity of FITC-N6NH2 and FITC-GUON6NH2 in RAW 264.7 cells and in mice. **(A)** Effect of different endocytosis inhibitors and low temperature on the uptake of FITC-labeled peptides in RAW 264.7 cells. Cells were preincubated with different endocytosis inhibitors (including amiloride, nocodazole, chlorpromazine, and MβCD, respectively) for 1 h before incubation with 10× MIC FITC-labeled peptides for 4 h. External fluorescence was quenched with 0.4% Trypan blue, and the fluorescence intensity was then measured by flow cytometry. CK is the negative control. N6NH2 was transported into cells through lipid rafts, but GUON6NH2 entered cells through macropinocytosis and microtubule polymerization. **(B)** Intracellular activity of N6NH2 and GUON6NH2 against *E. tarda* in RAW 264.7 cells. The ordinate showed bacterial reduction per mL of broth in RAW 264.7 cells after 18 h of incubation with peptides compared to the original inoculum. GUON6NH2 had higher intracellular activity against *E. tarda* than N6NH2 in RAW 264.7 cells. **(C)** Survival of mice. The survival rate of mice of 1 μmoL/kg N6NH2 was 80%, which was a little highter than that with 1 μmoL/kg GUON6NH2 (60%). Peptides have a higher survival rate than antibiotic. **(D)** Intracellular activity of N6NH2 and GUON6NH2 against *E. tarda* in the mouse peritonitis model. GUON6NH2 had higher efficacy against *E. tarda* than N6NH2 and norfloxacin in mice. (*) indicates the significance between control and treatment groups. *p < 0.05; **p < 0.01; ***p < 0.001; ****p < 0.0001.

To explore whether N6NH2 and GUON6NH2 can enter cells through endocytosis, several endocytosis inhibitors, such as amiloride (macropinocytosis inhibitor), nocodazole (inhibiting tubulin polymerization into microtubules), chlorpromazine (inhibiting clathrin-mediated endocytosis), and MβCD (inhibiting caveolin-mediated endocytosis) were used to treat RAW 264.7 cells. As shown in Figure 7A, the fluorescence intensity of the cells treated with MβCD and N6NH2 decreased by 21%, while amiloride and nocodazole reduced the cell entry rate of GUON6NH2 by 12.35 and 22.55%, respectively, suggesting that N6NH2 may be transported into cells through lipid rafts, while GUON6NH2 enters cells through macropinocytosis and microtubule polymerization.

### N6NH2 and GUON6NH2 Had Potent Intracellular Antibacterial Activity in RAW 264.7 Macrophages

The ability of N6NH2 and GUON6NH2 to eliminate intracellular *E. tarda* was evaluated in RAW 264.7 cells. As shown in Figure 7B, both N6NH2 and GUON6NH2 significantly reduced the proportion of internalized *E. tarda* cells and the inhibition rates of intracellular bacteria were over 99% in the peptide-treated groups. The killing efficacy of 1× MIC GUON6NH2 against *E. tarda* was over 97.07%, which was slightly higher than that of N6NH2 (96.82%). 5–50× MIC N6NH2 and GUON6NH2 killed all intracellular bacteria. This result suggested that GUON6NH2 has more potent bacteriostatic effects than N6NH2 against intracellular *E. tarda*.

### N6NH2 and GUON6NH2 Had a High Therapeutic Efficacy in Mice

To further elucidate *in vivo* antibacterial activity of peptides, the efficacy of N6NH2 and GUON6NH2 was determined in a mouse peritonitis model. The results showed the untreated mice began to die at 48 h after inoculation, and 80% were dead within 120 h. After treatment with 1 μmoL/kg N6NH2, the survival rate of mice was 80%, which was higher than that with 1 μmoL/kg GUON6NH2 (60%) and 1 μmoL/kg norfloxacin (40%) (Figure 7C). Moreover, for intracellular bacteria, the bacterial numbers in the blank control group were approximately 10^6^ CFU/mL; after treatment with 1 μmol/kg of N6NH2 and GUON6NH2, the numbers of intracellular bacteria were reduced by 1.54- and 1.97-Log_10_ CFU, respectively, higher than norfloxacin (0.35-Log_10_ CFU) (Figure 7D). Comparably, after treatment with N6NH2, GUON6NH2 and norfloxacin, the total numbers of bacteria decreased by 1. 45-, 1. 53-, and 0.49-Log_10_ CFU, respectively. These data indicated that the efficacy of GUON6NH2 against *E. tarda* in mice is superior to that of N6NH2 and norfloxacin.

## Discussion

*Edwardsiella tarda* can invade, survive, and proliferate in host cells (such as macrophages), leading to a high infection recurrence (Brett Finlay and Falkow, 1997). Traditional antibiotics cannot completely eradicate *E. tarda* due to poor internalization. A few vaccines of *E. tarda* are waiting to be translated into commercialized ones. Our previous study showed that the marine peptide-N6NH2 had a potent antibacterial activity against intracellular *Salmonella*, but it showed low stability to trypsin. In this study, to improve stability and activity, the novel derivatives of N6NH2 were generated by modification with natural/unnatural residues, their properties and intracellular activity against *E. tarda* were investigated in RAW 264.7 cells and in mice infected with *E. tarda*.

The stability of AMPs can limit clinical applications; particularly, pH and protease stability of peptides are important factors in oral administration of peptide drugs (Eckert, 2011; Zhang et al., 2011). In this study, N6NH2 and its derivatives retained their inherent antibacterial activity in a wide temperature (20–100°C) and pH (pH 2–10) range (Figures 1C,D). Moreover, DN6NH2 and GUON6NH2 were more potent resistant to trypsin than N6NH2, N6PNH2, and V112N6NH2, respectively (Figure 1E). GUON6NH2 displayed lower cytotoxicity and hemolysis (<1%) than other peptides (<5%) (Figures 1A,B). The time-killing curves showed that GUON6NH2 had a faster killing rate and stronger selectivity for *E. tarda* than norfloxacin and other derivatives (Supplementary Figure 2). Meanwhile, similar to N6NH2, GUON6NH2 (with the MICs of 2–8 μg/mL) exhibited more potent activity against Gram-negative bacteria than other derivatives (2–32 μg/mL) (Table 2); it may be related to an addition of N,N,N’,N’-tetramethylguanidine and Orn at N-terminus of peptides, which can increase the positive charge and improve antibacterial activity (Czihal et al., 2012). Comparably, the bacterial growth in the norfloxacin-treated group showed a slow reduction and the regrowth of *E. tarda* was observed at 10 h, which may be associated with the development of drug resistance.

The combination of AMPs with antibiotics is an effective strategy to combat resistant pathogens (Uckun and Qazi, 2019; Zharkova et al., 2019). Our results showed that the mean PAE for GUON6NH2 was 3.97 h, which was more than 2.65 times longer than that of N6NH2 (1.5 h), indicating a better bactericidal effect of GUON6NH2 against *E. tarda*, superior to norfloxacin (Figures 2A,B). Moreover, GUON6NH2 showed a synergistic effect with norfloxacin and chloramphenicol (Figure 2C). The results suggested that GUON6NH2 has great potential for biomedical applications due to its potent activity, high stability, and low toxicity.

It is generally accepted that some AMPs exhibit antimicrobial activity through cell membrane permeability (Yeaman and Yount, 2003). The peptides first aggregate on the bacterial surface, pass through the cell wall barrier, and then interact with the cell plasma membrane. When the local peptide concentration reaches the threshold that can produce an effect, the peptide changes in conformation and then inserts into the cell membrane, causing membrane potential disturbance; the membrane integrity is destroyed, or pores/ion channels are formed, which eventually leads to cytoplasmic leakage and bacterial cell death (Teixeira et al., 2012). In our study, GUON6NH2 could bind to LPS and penetrate the outer and inner cell membranes of *E. tarda*, superior to N6NH2 (Figure 3 and Supplementary Figure 3). SEM and TEM results showed that N6NH2 and its derivatives did not destroy the cell integrity, but caused the leakage of the contents and killed bacteria (Figure 4), which is consistent with the intracellular localization of N6NH2 and GUON6NH2 in *E. tarda* (Supplementary Figure 5). It indicated that N6NH2 and GUON6NH2 can target and disrupt the membrane, and they can enter the bacterial cells and kill bacteria, which is similar to other AMPs including buforin II (Conner and Schmid, 2003) and pyrrhocoricin (Brett Finlay and Cossart, 1997).

Macrophages play a key role in host defense against bacterial infection, and intracellular pathogens prefer to live in macrophages to proliferate and escape the innate immunity (Qin et al., 2014, 2017). *Brucella* is demonstrated to survive and replicate within membrane-bound vacuoles in phagolysomes (Leung et al., 2012). Likewise, *Salmonella* can replicate in membrane-bound compartments, *Salmonella*-containing vacuoles (SCVs), within mammalian cells (McGourty et al., 2012). It has been demonstrated that the mouse RAW 264.7 macrophages may be considered as a cell model for study interactions between *E. tarda* and macrophages (Qin et al., 2017). In this study, *E. tarda* a multiplicity of infection (MOI) of 1000:1 survived and replicated in macrophages with an increase of 7.36 Log_10_ CFU within 24 h (Figure 5A), which is inconsistent with previous reports in which the MOIs of 10:1 and 100:1 led to internalization and infection of *E. tarda* after a 2 h-inoculation period and *E. tarda* could replicate within vacuolar-like compartment in RAW 264.7 macrophages; this may be related to different infection time (Qin et al., 2017).

The premise of anti-bacterial drugs in cells to work is that the drug can reach its target and interact with bacteria (Carryn et al., 2003). In this study, N6NH2 and GUON6NH2 entered RAW 264.7 cells in a dose-dependent manner and were distributed in the cytoplasm (Figure 6). GUON6NH2 showed higher uptake efficacy than N6NH2 (Figures 5B,C), which may be related to different internalization pathways, including phagocytosis, macropinocytosis, clathrin-/caveolin-mediated endocytosis, etc. (Brett Finlay and Cossart, 1997; Conner and Schmid, 2003). The potential mechanism of cellular internalization of peptides is usually evaluated by using different endocytosis inhibitors (such as amilolide, norcodazole and chlorpromazine), which play a key role in selectively blocking specific endocytosis pathways (Lundin et al., 2008). In this study, N6NH2 was internalized in the cells through lipid rafts, while GUON6NH2 entered the cells by giant pinocytosis and cytoskeletal movement with microtubules involved (Figure 7A). Noticeably, GUON6NH2 exhibited higher intracellular bactericidal effect than N6NH2 in macrophages (Figure 7B) and in mice infected with *E. tarda* (Figure 7C), which was similar to peptide Api88 with N,N,N’,N’-tetramethylguanidine and Orn at N-terminus; the guanification of N-terminus and the amidation of C-terminus of peptide can result in increased positive charges, which can improve the antibacterial activity of peptides and stability in host cells (Zharkova et al., 2019). Moreover, there is growing evidence that the use of Orn as a charged moiety in peptides is preferable as the use of unnatural amino acids, which provides stability against proteases (Pieters, 2001; Laverty et al., 2011; Czihal et al., 2012; Schmidt et al., 2017).

In conclusion, four derivatives of N6NH2 were generated by an introduction of unnatural residues or substitution of natural ones. Among them, GUON6NH2 showed the best properties (including high stability and low toxicity) and antibacterial activity against *E. tarda*. GUON6NH2 had a stronger ability to bind to LPS and penetrate the outer/inner cell membranes of *E. tarda* than N6NH2. The two peptides could enter macrophages in a dose-dependent manner through endocytosis and distributed in the cytoplasm. GUON6NH2 had a stronger intracellular bactericidal effect against *E. tarda* than N6NH2 *in vitro* and in mice, respectively. Altogether, these findings suggested that GUON6NH2 may have great potential as a novel antimicrobial agent against infectious diseases caused by intracellular *E. tarda*.

## Data Availability Statement

The original contributions presented in the study are included in the article/Supplementary Material, further inquiries can be directed to the corresponding author/s.

## Ethics Statement

The animal study was reviewed and approved by the Animal Care and Use Committee of the Feed Research Institute of Chinese Academy of Agricultural Sciences (CAAS), and protocols were approved by the Laboratory Animal Ethical Committee and its Inspection of the Feed Research Institute of CAAS (AEC-CAAS-20090609).

## Author Contributions

HH, DT, XW, and JW conceived and designed the experiments. HH carried out all the experiments. RM, NY, and ZW guided the methods. ZW, TL, and NY prepared partial materials in the laboratory. YH contributed materials and reagents. HH and XW contributed to writing. JW contributed to funding acquisition. All authors contributed to the article and approved the submitted version.

## Conflict of Interest

The authors declare that the research was conducted in the absence of any commercial or financial relationships that could be construed as a potential conflict of interest.

## Footnotes

1 http://heliquest.ipmc.cnrs.fr/cgi-bin/ComputParamsV2.py

2 https://pepcalc.com/
