# Supplementary material for: Marine Peptide-N6NH2 and Its Derivative-GUON6NH2 Have Potent Antimicrobial Activity Against Intracellular Edwardsiella tarda in vitro and in vivo
Source: Front Microbiol. 2021 Mar 9;12:637427. doi: 10.3389/fmicb.2021.637427 (PMC7985170; doi:10.3389/fmicb.2021.637427)
Supplement: Supplementary file 1 [file Data_Sheet_1.doc]

**Supplementary Materials**

**Marine peptide-N6NH2 and its** **derivative-GUON6NH2 have** **potent antimicrobial activity against intracellular *Edwardsiella tarda* *in vitro* and *in vivo***

*Huihui Han^1,2^, Da Teng^1,2^, Ruoyu Mao^1,2^, Ya Hao^1,2^, Na Yang^1,2^,* *Zhenlong Wang^1,2^, Ting Li^1,2^, Xiumin Wang^2,3^* and Jianhua Wang^1,2^**

^1^ *Gene Engineering Laboratory, Feed Research Institute, Chinese Academy of Agricultural Sciences, Beijing 100081, People’s Republic of China,* ^2^ *Key Laboratory of Feed Biotechnology, Ministry of Agriculture and Rural Affairs, Beijing 100081, People’s Republic of China, ^3^* *Chinese Herbal Medicine Laboratory, Feed Research Institute, Chinese Academy of Agricultural Sciences, Beijing 100081, People’s Republic of China*

* Corresponding author

Prof., Ph.D., PI. Jianhua Wang and postal address of all authors as:

Gene Engineering Laboratory, Feed Research Institute

Chinese Academy of Agricultural Sciences, 12 Zhongguancun Nandajie St., Haidian District, Beijing 100081, People’s Republic of China

*E-mail address:* [wangxiumin@caas.cn](mailto:wangxiumin@caas.cn); wangjianhua@caas.cn

Phone: 0086-10-82106081, 0086-10-82106079; Fax: 0086-10-82106079

| Secondary  structure | N6NH2 (%) | | | N6PNH2 (%) | | | DN6NH2 (%) | | | V112N6NH2 (%) | | | GUON6NH2 (%) | | |
| --- | --- | --- | --- | --- | --- | --- | --- | --- | --- | --- | --- | --- | --- | --- | --- |
|  | H_2_O | SDS | TFE | H_2_O | SDS | TFE | H_2_O | SDS | TFE | H_2_O | SDS | TFE | H_2_O | SDS | TFE |
| Helix | 8.90 | 10.49 | 13.25 | 8.88 | 9.43 | 9.11 | 6.07 | 5.50 | 5.35 | 8.52 | 10.95 | 13.05 | 7.84 | 8.16 | 10.73 |
| Antiparallel | 15.17 | 63.52 | 26.42 | 19.45 | 17.78 | 18.60 | 69.59 | 2.22 | 3.96 | 30.36 | 14.77 | 32.33 | 1.04 | 0.59 | 10.73 |
| Parallel | 2.94 | 5.03 | 2.83 | 3.16 | 3.08 | 2.91 | 4.15 | 2.06 | 5.25 | 3.33 | 3.02 | 3.21 | 1.79 | 1.76 | 1.95 |
| Beta-turn | 30.23 | 5.72 | 35.28 | 28.33 | 29.10 | 31.49 | 6.94 | 38.00 | 9.60 | 23.21 | 29.85 | 26.29 | 39.25 | 35.66 | 45.04 |
| Rndm. coil | 42.76 | 15.08 | 22.21 | 40.18 | 40.62 | 37.79 | 13.25 | 52.22 | 75.74 | 34.57 | 41.41 | 25.12 | 50.00 | 53.82 | 31.56 |

**Table S1. CD spectra of N6NH2 and its derivatives in different solutions.**

**Table S2. Combination of peptide N6NH2 and GUON6NH2 with antibiotics against *E. tarda***

| Synergism | Peptides | |
| --- | --- | --- |
| Antibiotics | N6NH2 | GUONNH2 |
| Ciprofloxacin(Cip) | 0.3125 | 5 |
| Ofloxacin(Ofl) | 0.3125 | 0.625 |
| Enrofloxacin(Enr) | 0.3125 | 0.5625 |
| Norfloxacin(Nor) | 0.125 | 0.125 |
| Chloramphenicol(Chl) | 0.25 | 0.50 |
| Kanamycin(Kan) | 0.25 | 5 |


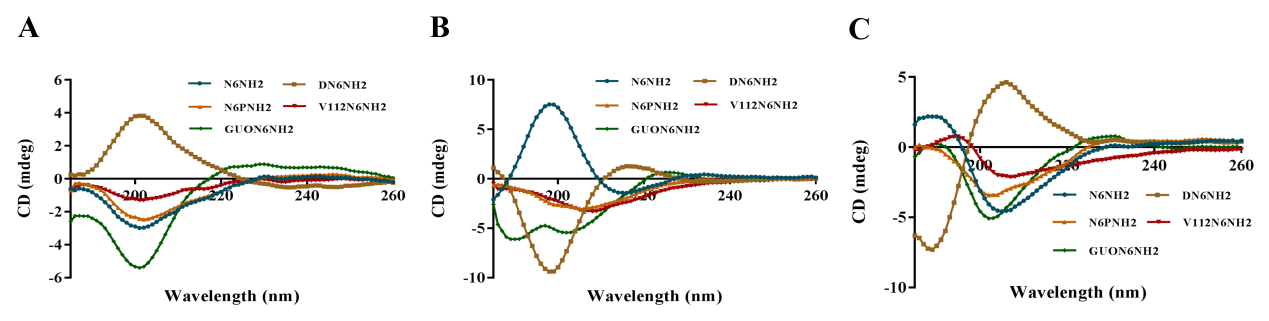


**Fig S1.** CD spectra of N6NH2 and its derivatives. (A) The secondary structures of the peptides in ddH_2_O. (B) The secondary structures of the peptides in 20 mM SDS. (C) The secondary structures of the peptides in 50% TFE.


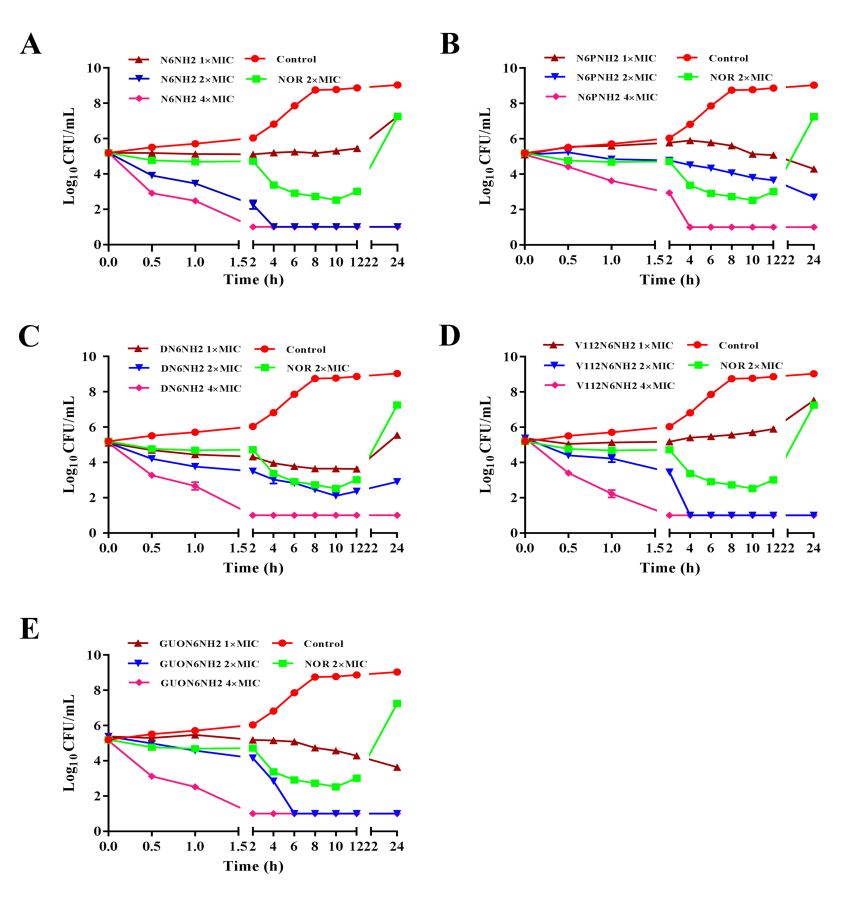


**Fig S2.** Time-kill assay of N6NH2 against *E. tarda.* *E. tarda* cells were treated with 1 ×, 2 × and 4 × MIC N6NH2 (A), N6PNH2 (B), DN6NH2 (C), V112N6NH2 (D), and GUON6NH2, respectively. The time-killing curves of peptides were plotted at different time intervals (0, 0.5, 1, 1.5, 2, 4, 6, 8, 10, 12, 22, and 24 h, respectively). The results are given as the mean ± SEM (n=3).


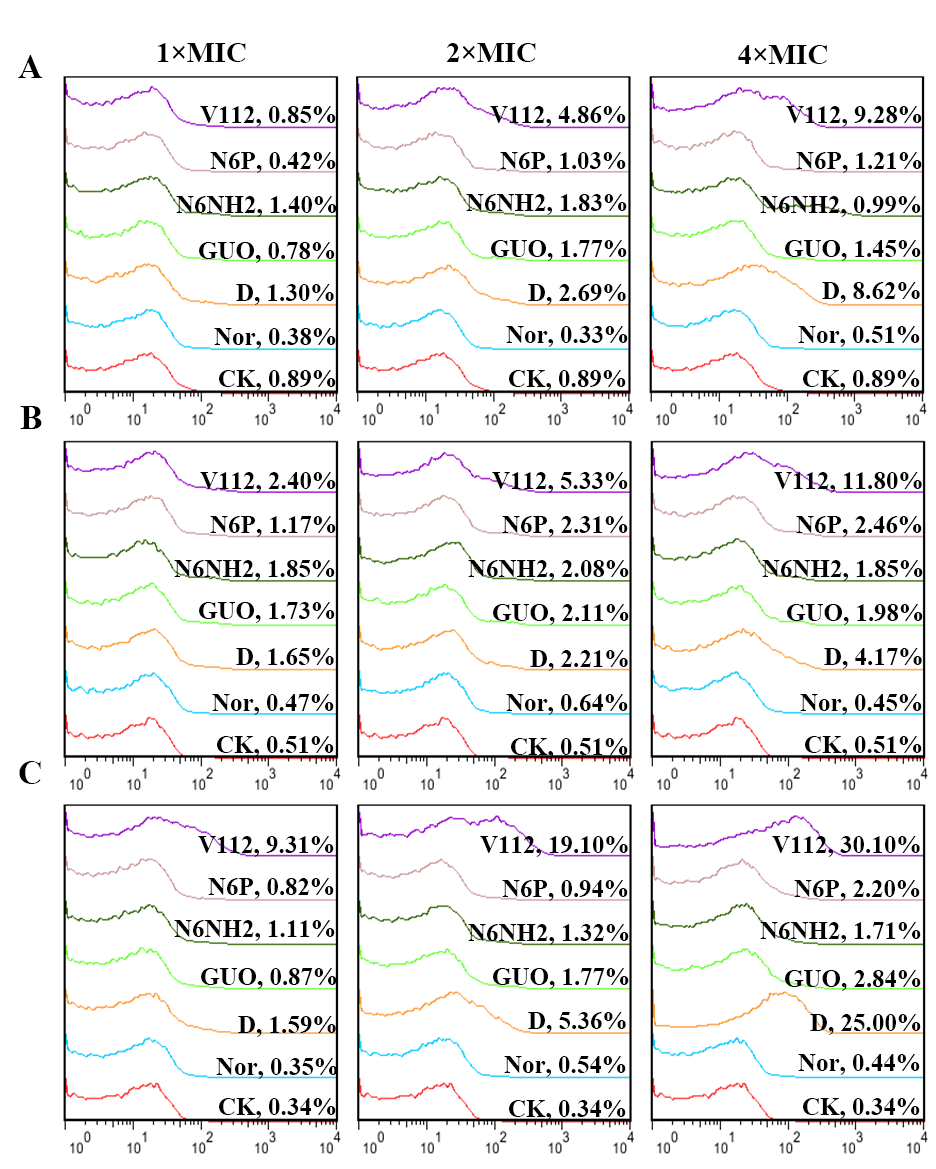


**Fig S3.** Flow cytometric analysis of PI-staining in *E. tarda* cells treated with the peptides, respectively. (A) treatment with peptides for 5 min; (B) treatment with peptides for 15 min; (C) treatment with peptides for 30 min. Nor, D, GUO, N6P and V112 indicate norfloxacin, DN6NH2, GUON6NH2, N6PNH2, and V112N6NH2, respectively.


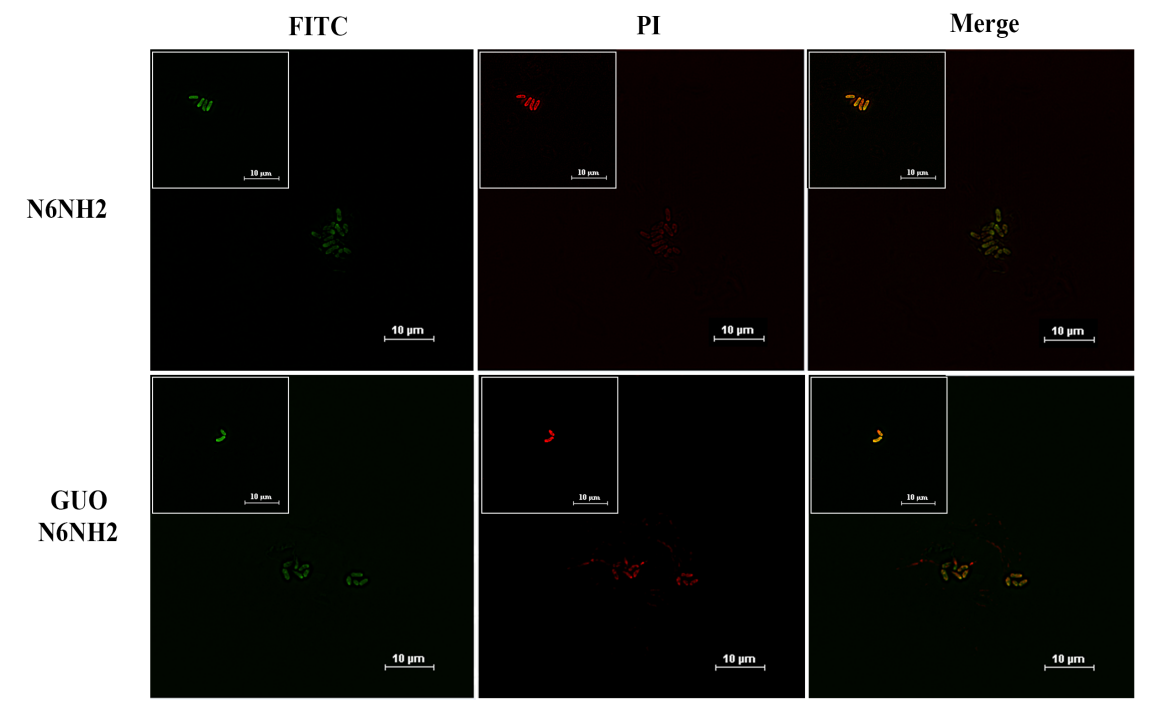


**Fig S4.** Optical DeltaVision OMX 3D-SIM images of *E. tarda* after treatment with FITC-labeled N6NH2 or GUON6NH2 and nucleic acid stain PI. From left to right, the images in columns show green signals from FITC peptides, red signals from PI and merged images.


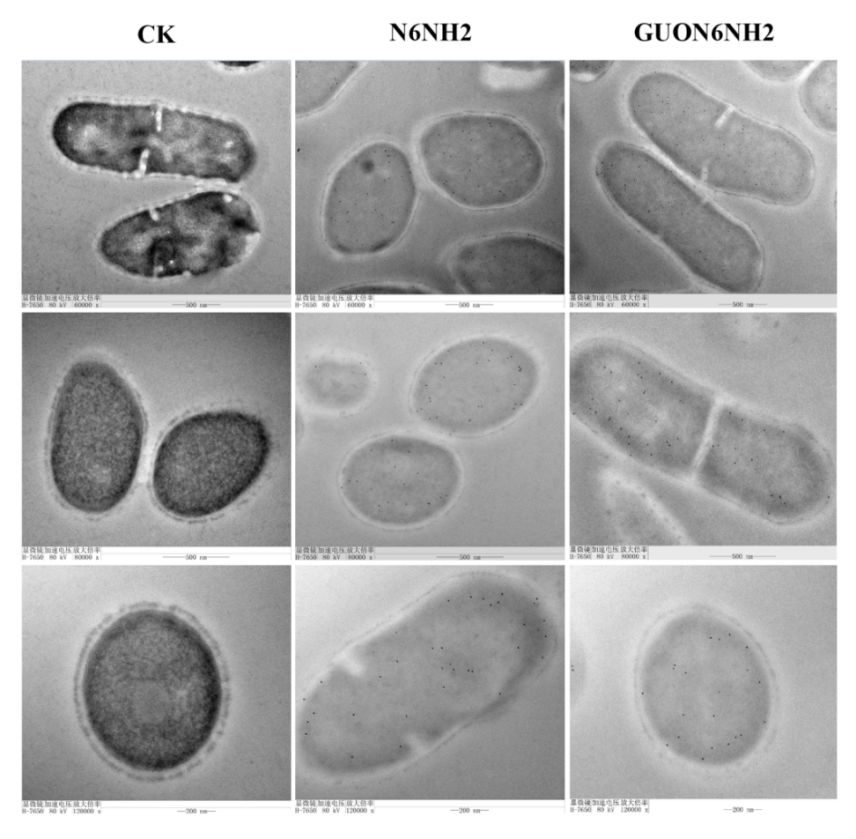


**Fig S5.** Immuno-TEM images of *E. tarda* treated with biotin-labeled N6NH2 and GUON6NH2. Images indicate 60000 ×, 80000 × and 120000 × magnification from top to bottom.
